# Medical and Physiological Commentaries

**Published:** 1841-04

**Authors:** 


### Art. V.

*Medical and Physiological Commentaries.* By Martyn Paine, m.d., a.m. —*New York,* 1840. Two vols. 8vo, pp. 1531.

Although we do not subscribe to the ancient proposition in all its generality, that " a great book is a great evil," there are certainly many instances in which it is true; and we are sorry to say that we are obliged to consider the work before us as one of the number. The multiplicity of subjects with which the mind of a medical man, who attempts to keep pace with the progress of his science, is constantly occupied, renders it desirable that ideas which are intended to be of practical utility to him should be submitted to his grasp in the smallest compass and most defined form, of which they are capable. For their merits in these respects we formerly bestowed high praise on Dr. Holland's Medical Notes and Reflections; a work to which the one now before us bears some degree of resemblance, being a collection of essays upon disconnected subjects, the result of the author's reading and experience. But here the resemblance stops. We compared Dr. Holland's short but lucid reviews to sketches drawn by the hand of a master; and, though mere outlines, not only embodying just and vivid conceptions, but evincing more power and even more learning than many an elaborate picture on which all the minor resources of art have been expended. To the latter part of this comparison Dr. Paine's work exactly answers. Of the degree of elaboration our readers may judge, when we say that Dr. Holland's single volume of six hundred pages contains thirty-four essays, whilst in Dr. Paine's bulky tomes we find but nine; and yet we hesitate not in asserting that any one of Dr. Holland's sketches will convey a greater number of clear ideas to a reader of ordinary capacity than the most highly-wrought of Dr. Paine's finished compositions. The reason is simply this,—that the latter contains a mass of materials, giving evidence, indeed, of great learning in the collection of them, but displaying an uncommon want of sagacity in their arrangement; so that the picture (to follow out our metaphor) is so perplexed by the fullness and disjointedness of its details, that it leaves no distinct impression on the mind.

A perusal of Dr. Paine's work has impressed us with a great respect for the author's industry and zeal; but we fancy that his mind must be deficient in one qualification, which is rather important to any one who sets up as a philosopher, (a title of which we should judge, by the heading of four of his essays, that the writer is rather ambitious,) namely, the capability of perceiving accurately the relations of ideas. This notion we have formed from the multitude of instances of loose and incoherent reasoning, of contradictory statements, and of misinterpretations of the opinions of others, which we have detected in his treatises. To bring a single objection against the assertion of another—whether that assertion be the statement of a fact or the enunciation of a principle—seems in his mind quite sufficient to overturn it. To show that a certain law is not capable of explaining all the phenomena of a given action appears to be regarded by him as quite sufficiently proving that the law had no bearing on the action. Now, any one who has given the slightest attention to the science of life—Biology—must be aware how little any phenomena exhibited by living beings can be attributed to the undisturbed operation of any single law; and how constantly the physiologist and pathologist are obliged to make allowance for circumstances which interfere with the operation of the simplest and best understood principles. But, it may be asked, does not the author lay himself open to similar attacks? Are his doctrines more securely defended than those of others? In his own estimation they certainly are; but we must take leave to differ from him. The foundation of his system is the attribution of every phenomenon which cannot be *fully* explained in any other way, to the undivided operation of the "vital forces;" a sort of "independent company," of which the "vital principle" is the commander. Any attempt to penetrate their ranks is useless; their shields form an impassable barrier, and their spears defy the invader. But ancient armour is of little avail against the resources of modern warfare; a few cannon-balls sweep the supports of these old-fashioned soldiers from under them, and the invaders boldly make their way into the guarded spot. To speak without metaphor, we consider that any attempt to check the course of enquiry, by thus attributing all unexplainable or unexplained phenomena to the immediate influence of that mysterious agent, the "vital force," is a decided retrogression in physiological science. For there can be no doubt that many of these phenomena are, to say the least, partly explicable on physical principles; and that those which result from the vital properties of organized bodies still take place under the government of fixed laws, which it is the legitimate province of the philosopher to investigate.

It seems to us as if Dr. Paine's mind—possessing, as it evidently does, much power, but, unfortunately, misdirected in the application of it—would have been much benefited by that training in the school of physical science, which would have given him habits of more correct reasoning and a clearer perception of the bearing of general principles. The latter with some persons is intuitive, by others it is acquired: from whatever source it arises it is one of the chief characteristics of a truly philosophic mind. We cannot better illustrate the deficiencies and errors we have laid to Dr. Paine's charge than by referring briefly to his "Appendix on the Microscope." (vol. i. p. 699.) The extended use of this instrument "threatens," in his opinion, "a subversion of physiological science." He forgets that, faithfully employed, it can only disclose *facts*. But facts are stubborn things. He quotes a number of instances in which microscopic observers have differed,—as, for example, the size and form of the globules of the blood—the structure of the nervous tubules; but he does not tell us how completely, with instruments much improved during the last few years, and with increased knowledge of the circumstances which can affect the accuracy of the observation, all good microscopists are now agreed on these as upon a multitude of other interesting and important questions. "Finally," he concludes, "our microscopical philosophers would have it, that all nature may be ultimately resolved into microscopical *insects* (!) The very rocks are composed of them—nay, the earth itself..... *On weighing one of their shells*," he afterwards tells us, "it was found to be the 187,000,000th part of a grain;" a most unfair distortion of an *estimate* founded upon measurement of the size of these curious bodies, and the known weight of collections of them containing a number which may be calculated by their size. In upholding this ridiculous doctrine, he tells us that "Professor Hitchcock is alone in his glory in the western hemisphere." We are assured, however, on the authority of Dr. Paine, who does not mention having examined the objects for himself, that "the objects mistaken for fossil animalculæ are nothing but crystalline spiculæ of earthy or metallic substances." When Dr. Paine has taken the trouble to examine, as we and hundreds of others have done in this country, the living animalcules, and the beautifully-organized siliceous cases which accumulate as a kind of mud at the bottom of the water in which they breed, and has compared these with similar remains, which, preserved in the most complete perfection, constitute the Polierschiefer of Bilin, or the mealy siliceous earth used as food by the Laplanders—he may perhaps regret that he has hazarded his credit upon so rash an assertion.

Dr. Paine's First Essay is devoted to the consideration of the subject of vitality; and in this, says our author, " we have endeavoured to show that the great question relating to the vital powers is in no respect a speculative one; and since all our other subjects revolve about it, we have made that article of unusual extent." It professes, indeed, to comprise a *full* discussion of the whole subject; but when we say that Dr. Prichard's luminous treatise is not once referred to, that Dr. Fletcher's chapters on the subject have received scarcely any attention, and that the lengthy discussion of Dr. Carpenter's views is based only on the extracts from his work which Dr. Paine found in our review of it, our readers will have an opportunity of judging whether it can be regarded as a *complete* one. He quite prevents Dr. Carpenter from complaining, however, of this kind of treatment, by the following preliminary apology. " We notice Dr. Carpenter's opinions, without the advantage of reading his work, on account of his high reputation and the encomiums of his able reviewer." We rather suspect, however, that Dr. Carpenter would have been very willing to forfeit the compliment, for the sake of having his views either fairly represented, or passed altogether without notice. On a subject of this abstract nature, where so much depends upon the particular sense in which terms are employed, and where passages taken altogether out of their connexion are so liable to be misinterpreted, a critic cannot be too careful to take an accurate survey of the whole of his author's argument, before running foul of any particular portion of it. This we have endeavoured to do in regard to Dr. Paine; and we must take the liberty of saying that if we do not give a fair representation of his views, it is because they are expressed in such vague and often contradictory phraseology, that we are not always certain of his meaning. We shall establish this claim to his indulgence, before going further.

" Returning to our enquiry" says Dr. Paine (p. 12), " ' what is life?' and to the consideration of its definition by Bichat, and the philosophers of his school, we consider the *functions* as being merely the result of peculiar forces operating upon organic matter, and that *life* virtually consists in the coexistence of these forces and that peculiar substratum. The forces are, to a certain extent, in a passive state, when not excited by their appropriate stimuli. But they are still the essence of life; and whilst they endure, whether in an active or seemingly passive condition, life is constituted." Now here, Dr. Paine evidently recognizes the existence of a " force" as something distinct from matter, on which it acts. In the previous paragraph, he speaks of " force" and " property" as synonymous; and we are thus enabled to understand the idea which *he* attaches to the following definition, contained in a subsequent part of his essay. " The foregoing considerations," he says (p. 19), " would lead us to regard life as a cause; and to define it as consisting of certain specific properties appertaining to organized matter, which are more or less capable of resisting the destructive agencies of inorganic matter, and the forces to which it is liable, and of protecting against them the matter in which they are inherent. . . . We thus," he adds with considerable selfgratulation, "embrace the elements of a good definition; philosophical accuracy, brevity, and a peculiar universal characteristic."

We do not know when it has been our fortune to meet with a more complete jumble of ideas, than that which would be involved in the foregoing sentences, if interpreted according to the usual meaning of the words composing them. By philosophical writers on any department of science, the term *force* is only applied when an action of some kind is taking place; thus the force of gravity, that is, the attraction subsisting between the earth and the falling body, causes the clock-weight to descend; or, if checked, occasions a pressure against the impeding substance. We cannot ourselves conceive of a force as having an existence distinct from the matter which manifests it. The Creator, in giving origin to that which we term matter, by that very act created the forces by which different material bodies operate upon one another. For all forces result from the action or operation of the properties (thus differing essentially from the properties themselves) with which matter is endowed; some of these being common to all forms of matter, whilst others are restricted to certain kinds of it. Thus a magnet has the *property* of attracting iron; but no *force* exists, until a piece of that metal is brought within the sphere of its attraction. To speak of a "passive force" is therefore a contradiction in terms. What should we think of a person who should talk about a "passive force" existing in water, by which are effected the mighty operations of the steam-engine? Does any *force* exist until the water has been converted into steam by the *stimulus* of heat, and the steam by a still further addition of temperature caused to expand with violence? Assuredly not. Yet this is only carrying out Dr. Paine's doctrine in the mode he himself warrants, when he tells us (p. 10), that the Deity is *not* the immediate cause of the phenomena of nature, but that he has brought into existence a number of *forces* (which he compares in their operation on matter to the mind, and which must, therefore, be so many separate *entities*) which are the proximate causes of all phenomena. Though the advocates of the vital principle have clung to this doctrine, for the purpose of explaining more readily (as they imagined) the phenomena of life, we certainly never expected to see it revived in the nineteenth century, in its application to material operations in general, by a writer of Dr. Paine's pretensions, and in a country where novelty is so eagerly grasped at. We cannot help imagining that Dr. Paine mistook this hypothesis for a new one; but he will find it to be the one which was adopted in the very infancy of philosophy, by those who endeavoured to account for the phenomena of nature.

Having disposed of *forces*, we will turn for a short time to *properties*. And we would ask, what other notion of matter do we possess than that derived from those properties which either directly affect our senses, through the material changes they produce in our organs, or which, by the influence of different bodies on one another, give rise to forces that produce phenomena of which we in like manner take cognizance? Subtract from *our notion* of matter all those properties by which we characterize it, and what remains? Nothing. On the other hand, we may ask, *if it is possible* for the properties to be separated from the matter itself? Can they have a distinct existence? Can we conceive of the quality of hardness, or whiteness, or smoothness, without some material body to which we attribute it? Or, to put the question in another form, can we conceive of an adjective as having any force without a substantive, and is not the latter always understood when the former is used alone? We hope that few of our readers are so bewildered by speculative abstractions as to be unable to use their common sense in answering these questions.

Having enlarged so much upon these preliminaries, a very brief statement of Dr. Paine's views on the subject of life will suffice, and this we shall give in his own words: "The more, therefore, we investigate the subject, the more are we satisfied that life consists in the integrity of the vital properties associated with organized matter; that the vital actions are only the results of life, or of the foregoing conditions; that the vital properties or forces are essentially distinct from organized matter itself; and that organized structure may exist entire without the properties of life, although the former is necessary to the existence of the latter." (p.29.) Moreover, at the commencement of the essay, we are given to understand that *vital principle* is employed only as "a collective term, referring to the universal cause of animal and vegetable life." Now it surpasses our comprehension to understand how that can be legitimately stated to be *a cause*, which implies the conjoint action of a number of causes. As far as we can understand Dr. Paine, all the vital properties, as we should term them, appertaining to organized matter, as, for instance, contractility and sensibility, are to be regarded as distinct existences, the combined operation of which produces the phenomena of life. Thus, we have not *one* vital principle (using that term in its ordinary sense) but many. Further, he tells us in one breath, that the "vital properties or forces are essentially distinct from organized matter itself," and that they can go away and leave this matter in a state of perfect integrity, whilst in the next he informs us that they cannot exist without it. What becomes of them, then, when they leave the organized body to shift for itself? If they have an existence so essentially distinct as to be capable of leaving it, it stands to reason that they must be capable of existing without it, yet Dr. Paine says they cannot. A writer who can so contradict himself scarcely needs to be exposed by us; and we should not think it worth while to waste more words upon this Essay, were it not that he has so strangely misunderstood and misrepresented our own views. As on this subject we formerly expressed our complete accordance with Dr. Carpenter's positions, whilst differing from him on other points, we shall in our present observations identify our own with them, more especially as Dr. Paine's criticisms are directed to both alike.

Dr. Paine accuses Dr. Carpenter of a want of consistency, in applying physical and chemical laws to the explanation of some of the phenomena exhibited by living beings, whilst he attributes others to the vital properties of their organisms. It will be hereafter seen that Dr. Paine, with most laudable zeal in defence of the principles he professes, defies the whole array of chemists to prove that *any* of the changes concerned in digestion, respiration, or the other functions of the living body, are in the least within their province. We cannot, therefore, be surprised at his accusation, but we apprehend that it will find few supporters. No physiologist who has thoroughly investigated his science can, we think, hesitate in the belief, that there are many phenomena in which the laws of vitality are only remotely concerned, and in which the immediate operation is dependent upon principles recognized elsewhere. To what extent we can carry such explanations is certainly a matter of speculation only; but that they are applicable in general to the changes of composition taking place in the organism, we think that the recent progress of organic chemistry (of which Dr. Paine seems but little aware) most distinctly intimates. Neither Dr. Carpenter nor ourselves ever advocated the doctrine that *organization* could be the result of any chemical or physical laws; on the contrary, Dr. Carpenter most distinctly repudiates any such idea, and shows that these laws can only operate in the preparation of those *organizable materials*, which, until organized, do not exhibit any vital properties. That the phenomena exhibited by living beings are influenced by many concurrent causes, is a doctrine to which no one in his senses can refuse his assent. Will Dr. Paine tell us that the particles of their organisms are, as soon as they are brought under the laws of vitality, removed from the influence of gravitation? that the heart forces the blood through the arteries according to any other laws than those which would govern the motion of a fluid of similar consistence, when impelled by a pressure similarly applied by artificial means? Then why should we stop short, and say,—the operation of chemical laws is suspended, whilst that of physical laws remains in force? In almost all sciences there are phenomena which general laws *appear* inadequate to explain; but the philosopher is not disposed to consider them as altogether beyond the pale of these laws, unless he can show that they are governed by different principles; and he trusts to time and further knowledge of the circumstances which modify their application for the solution of the difficulty. Who, for example, would attribute to a *meteorological principle* the varied phenomena which at present baffle the philosophic skill of the most profound enquirers to explain? Now we hold that, upon the Newtonian axiom so universally admitted, which forbids the construction of unnecessary hypotheses, it is the part of the philosopher to refuse his assent to any new doctrine of *vital affinities*, or of the subversion of chemical affinities by vitality, or any other equally vague speculation. It appears to us that, to entitle such a doctrine to assent, it would be necessary to prove that oxygen, hydrogen, carbon, and nitrogen, and the other elements of organized structures, follow, in the living body, laws of union so entirely different from those which control their inorganic combinations, that, when brought together under the same circumstances in respect to all those influences (for example, form, temperature, state of combination, state of division, electric condition, &c.) which the inorganic chemist recognizes as affecting the result, the influence of vitality *directly* exercised upon one shall entirely change the product. In this case, oxygen would cease to be oxygen, for it would lose those properties by which we know it, and upon which alone (as we just now endeavoured to show in regard to matter in general) our notion of it is formed. Now Dr. Paine tells us (vol. ii, p. 120, *note*,) that the laws regulating vital affinities are "more clearly established than any in chemistry." We would suggest to him to make an exposition of them the subject of his next work, since nothing can give him a higher rank as a physiologist than such a distinct statement of them as may rank with the Daltonian laws of definite proportions. Until, however, the proof which we think we have a right to require is brought forward, we shall rest upon the fact, now fully established, that, by influences acting on chemical principles, one organic product (not an organized tissue) may be converted into another, as distinctly indicating that the elements of all these products are held together by no other than chemical affinities.

The doctrine which Dr. Carpenter has propounded respecting vital properties, and which is essentially the same as that upheld by Dr. Prichard, Dr. Fletcher, Mr. Roberton, and other able writers on the same side, may be concisely stated as follows: Certain forms of matter (especially oxygen, hydrogen, carbon, and nitrogen,) are endowed with properties which do not manifest themselves either in these elements when uncombined, or in those combinations of them which the chemist effects by ordinary means. But they do manifest themselves when they are united into those peculiar compounds which are known as *organic*, and when these compounds have been submitted to the process which is termed *organization*. It is possible that the first of these conditions may be imitated by the chemist, but the last can only be effected by a previously-existing organism. We assert, then, that the very act of organization causes the materials acted on to exhibit properties *quite distinct* from those ordinarily termed physical and chemical, which properties cannot be caused to manifest themselves in any other way than by the series of operations just described. We cannot see in what points this doctrine is open to objection. No one can say that the properties *do not* exist in a dormant state because they do not manifest themselves to him. The answer to such an assertion would be simply this: Put the matter in question into the conditions specified as necessary, and you will see the operation of the properties. And this is but following out the mode of reasoning which is constantly employed in physical science, for no properties of matter but those which directly affect our senses can be shown to exist in any form of it except by affording it certain conditions for its manifestation. For example, how do we know that oxygen is a supporter of combustion, except by trying it with a combustible substance? Or how do we know that magnetic properties may be made to show themselves in iron, until we have placed the metal in the necessary relations with a magnet? How, then, can we expect to find contractility or sensibility in any combination of oxygen, hydrogen, carbon, and nitrogen, until it has been converted into an organized tissue by a previously-existing organism? And have we any more right to say that "vital properties," or "vital forces," or a "vital principle," have been *superadded* in the last case, than to assert that "magnetic properties," or "magnetic forces," or a "magnetic principle," have been *superadded* in the former one?—a mode of expression which, if it is to mean anything like that which the words import, no enlightened physical philosopher would think himself justified in employing.

From this explanation it will appear that a large part of the criticism which Dr. Paine has lavished upon us for attempting to explain the phenomena of life upon physical and chemical principles is entirely misdirected; since, whatever difference of opinion there may be as to those changes of composition which are concerned in the formation of organizable materials and the products of secretion, there is none whatever as to the fact of these materials, when organized, being endowed with properties entirely distinct from any which can be traced in inorganic matter. Whence these properties are derived is the point on which we disagree: Dr. Paine maintaining that they hover about with a kind of undefined existence, ready to enter organized tissues when ready for their reception; whilst we argue that they were as much present in the elements as any of their other properties, which only exhibit themselves in certain conditions, and that we have no more right to deny their presence there than we have to assert that a sleeping man does not possess a mind, because it does not show itself in his actions.

It would occupy by far too much of our time and space if we were to follow our author through all the weak points of his lengthy disquisition. But we would just remark that, as, according to all the rules of philosophising, the *onus probandi* rests upon those who maintain the existence of a "principle" which others declare to be unnecessary, it is for the advocates of a "vital principle," or of "vital properties distinct from organized tissues," to show that an organized body which has suffered death by the supposed departure of these but which *retains its entire organization*, can be subjected to the action of the ordinary vital stimuli, without exhibiting vital actions. We take the liberty of denying altogether the possibility of this; and assert that death can never take place without some important change in organization. Let our opponents prove the contrary. Further, we assert that in all cases in which an organized body, such as an egg or seed, exhibits a vital power of resisting the decomposing effect of external agents, that power is due to some amount of vital *action* going on in it; and it is for our opponents to prove that no such action is going on. For instance, when Hunter found that a fresh egg was longer in freezing than one whose vitality had been previously destroyed, we assert that the effect was due to the respiration which was taking place in the former, and which retarded its cooling; for, by another experiment of Hunter's (On Animals producing Heat, experiment xxvii.), we learn that, under the hen, the temperature of the living egg is two degrees higher than that of the addled one. We therefore feel no doubt that the difference in the power of resisting cold possessed by a living and a dead egg is to be accounted for on precisely the same principles as that which may be observed between an hybernating and a dead animal. The respiration is feeble; but it is in itself an important process, and indicates the continuance of other changes within the system. The power of resisting spontaneous decomposition may be accounted for on the same simple principles; and it is for those who seek for new ones to show that they are necessary. This Dr. Paine has by no means done; and we therefore now take leave of his exclusive system of vitalism until it shall come before us on some better foundation.

The vagueness of our author's views in reference to the "vital forces," and the complete inutility of them in practice, is well shown in the Second Essay on the Philosophy of Bloodletting. After quoting sundry opinions on the *modus operandi* of bloodletting as a therapeutic means, he gives us his own conclusion, that it acts by producing an "impression on the vital forces." Now we should like to know what is gained by this form of speech, for it is nothing more. We thought that at first Dr. Paine was intending to explain the nature of this impression; for he tells us that the loss of blood produces, as its first effect, a contraction of the blood-vessels, which is propagated by continuous sympathy towards the heart on one side and the capillaries on the other, so that the action of the one is modified and that of the latter restored to its normal condition. This doctrine, by the way, precisely corresponds with that of Dr. Macartney; and though Dr. Paine elsewhere ridicules his phraseology, he employs something so very like it in this instance that we can scarcely overlook the parallel. The "alarm felt by the extreme vessels and capillaries," according to Dr. Paine, must be tolerably like the "sense of danger" which Dr. Macartney attributes to them; and, so far as we can discern the general theory of the former through the cloud of words in which it is enveloped, it may be concisely stated in the following proposition set forth by the latter: "The intention of drawing blood from the system is to produce that kind of impression which is followed by a weaker action of the heart and a more contracted state of all the smaller arteries in the body, and by that means inflamed parts are included in the general condition which precludes the possibility of inflammation." (Macartney on Inflammation, p. 151.) But it would seem that Dr. Paine, like Dr. Macartney, really considers the general impression upon the vital powers as the immediate cause of the contraction of the vessels; for just after showing how this change is propagated by sympathy, he tells us that "a victory is obtained over the disease" (inflammation) even before the blood is expelled from them, so that we do not seem to have advanced anything; and are, after all, to rest satisfied with the "direct and efficient impression" of bloodletting "upon the *vires vitæ* of the capillary vessels," as an ultimate fact. Let those esteem it as such who think it of any use to them. And in this way, Dr. Paine endeavours to satisfy himself and his readers, in regard to the influence of particular forms of the remedy. "From the foregoing considerations, we infer that the peculiarities of leeching are owing to some specific impression exerted by this remedy upon the forces of life, which no other mode of abstracting blood can directly establish." Are we one whit the wiser after all this learned discussion than we were before?

Of the practical portion of this essay, a large part is devoted to a critical review of Dr. M. Hall's opinions on the subject, to which our author strenuously objects. To the general merit of these opinions, we have on a former occasion borne a willing testimony; and our appreciation of their value is but little affected by Dr. Paine's objections. It is well known to every practitioner that no principle of treatment is without its exceptions; and Dr. Hall is as ready as any one to admit that this is the case with regard to the rules which he lays down. Dr. Paine brings together all the evidence he can gather in favour of the position he adopts, that the lancet is not used with sufficient freedom; and he seems almost a stranger to those distressing *sequelæ* of excessive loss of blood, which few practitioners in this country do not frequently witness. Believing that, whatever may be Dr. Paine's qualifications as a philosopher, he is a man of correct and extensive observation (on subjects, at least, on which his prejudices allow him to use his sight and his sense), we can only justify the very free and all but universal recourse which he advises to the use of the lancet, by supposing that his countrymen, young and old,* have much more stamina than the original stock—beef-eating and plethoric as it has been usually accounted.

We are inclined to think that he especially needs enlightenment as to the prevailing constitution of the poor of our large cities; where deficient and unwholesome food, unmitigated exposure to a most impure atmosphere, extreme filth of the person, depravity and wretchedness of mind, and all the other evils of a squalid poverty which can scarcely exist in anything like the same extent or degree in any part of the United States, not only combine to depress the vital powers of those immediately affected, but, as Dr. Alison has recently well shown, exert a most unfavorable influence on the health of the whole community. In regard to the use of bleeding in fever, too, we scarcely think that Dr. Paine has given sufficient weight to the character of the epidemic. In places where fever is constantly prevalent, such as Edinburgh, the change of type manifests itself fully as much by the kind of treatment found to be most successful, as by the alteration of the symptoms; and a few months will often reverse the therapeutic system as completely as if a new disease had appeared.

The next essay, which seems to us still more vague in its object and tedious in its character, is on the "Humoral Pathology;" the resurrection of which from its state of oblivion seems to excite so much alarm in Dr. Paine's mind, that nothing but a manifesto of 300 closely-printed pages can relieve him of the weight of responsibility, which, as the champion of neglected truth, he believes to rest upon himself. The best attention which we have been able to give to this essay has failed to impress our minds with a clear idea of the author's opinions upon its subject. The expressions in which they are couched are often so contradictory (to our minds at least), that we know not which to adopt. Take for example the following consecutive sentences: (1.) "These considerations will show us that the blood is neither a primary cause of disease in the solids, in virtue of its own morbid condition, nor can it be an aggravating cause of disease when altered in its character by the morbid action of the solids. (2.) None will deny what is affirmed by M. Andral, that every morbid change in the action of the solids is probably followed by a change in the blood. (3.) Whilst we fully agree with him, that any *primary* alteration of the blood of a morbid nature must, with greater certainty, produce disease of the solids. The latter proposition is the basis of humoralism." (p. 646.) Now the first of these sentences appears to us so far contradictory of the third, that we can only get out of the difficulty by supposing it to be our author's meaning that no primary morbid alteration *can* take place in the blood—an opinion so completely opposed to all that sound physiology and pathology teach us, that we scarcely venture to attribute it to him. As far as we can understand him, he believes disease to consist in a deranged action of his independent vital powers or forces, to which all material changes are but secondary. As we have already attempted to show that these forces have no existence but in the actions of the material organism, depending upon the properties with which matter was at first endowed by its Creator, we

* The following is one of Dr. Paine's general conclusions: "Bloodletting is equally safe at all periods of life, is most indispensable in old age,! though not less important in many diseases of infancy."

need not here stop to discuss the unphilosophical character of this hypothesis. In regard to the main question, we may briefly state our opinion:—that the *vital* properties of the blood (for with such we have formerly shown it to be endowed) are, like its physical properties, capable of alteration by various causes; that some of these causes may originate in the system itself—as, for example, a disorder of the apparatus of sanguification, of assimilation, of respiration, of secretion, whilst others are external and act directly on the blood, especially through the medium of the respiratory organs and the nervous system; and that, when the quality of the blood has been changed, either primarily or secondarily, this alteration may give rise to other morbific changes, and will especially affect what is commonly termed the "constitutional state."

The Second Volume opens with a long essay upon Animal Heat; in which the author, in pursuance of his favorite doctrines, endeavours to rescue this function from the grasp of the chemists, and to prove it to be an undoubted offspring of pure vitality. What is his success, we shall leave it to his readers to determine; for to go through his whole train of reasoning on the subject would occupy too much of our space. This we the less regret, as it appears to us extremely inconclusive. But of his general results and of the mode in which he attains them, we shall adduce a few specimens. "The only connexion which has been reasonably shown," he observes in commencing, "of the forces of chemistry with the process of respiration relates to the decomposition of the air. From this, and from the necessity of respiration to animal temperature, in common with the other products of vital actions, has followed the induction that the generation of heat is a chemical process." (p. 4.) We will not impute the mistake of calling the separation of the oxygen of the atmosphere from the nitrogen *mixed* with it an act of *decomposition* to Dr. Paine's ignorance of the meaning of chemical terms; but we assert that a much closer connexion has been shown between the act of respiration and the laws of chemistry than he states. For not only is oxygen removed from the atmosphere, but carbonic acid is added by this process; and to mention one only of the proofs that this exchange follows ordinary chemical laws, it takes place out of the body as well as in it, under the influence of purely physical conditions. Again, Dr. Paine assumes that the production of animal (or rather organic) heat is not more intimately connected with respiration than are the other functions; slurring over the important experiments of Newport on the production of heat in insects, in which this relation is shown to exist in a remarkable degree, and neglecting altogether the results adduced by vegetable physiologists as to the constant relation between the development of heat, in those flowers which most prominently exhibit the phenomenon, and the amount of oxygen converted into carbonic acid. The case stands simply thus: oxygen is introduced into the system from the atmosphere. In some part of the system (*where*, for the general argument is of no consequence), it combines with carbon, set free in the ordinary processes of nutrition; and in this state it is thrown off in the gaseous form from the body. Now will Dr. Paine tell us why, since we know that the combination of carbon and oxygen elsewhere produces heat, it should *not* do so here? And if it does—and if, with a few occasional *apparent* exceptions to which the operation of all physiological laws is subject, the general relation between the amount of heat generated and the amount of carbon united with oxygen is constant—we ask, why should we seek for any other efficient cause? But, when we apply the question a little more closely, it is seen that chemists have changed the position which they occupied some years ago; and, instead of maintaining that the union of the two elements takes place in the lungs, they consider it as diffused over the general system. So, says Dr. Paine, like a victorious general, "the chemists having abandoned this old entrenchment, we shall take possession and fortify it with the forces of life." But Dr. Paine seems to forget that it was not by *his* army, to use his own metaphor, that the chemists were compelled to yield; but by a division of the more enlightened among themselves, who saw that this new position would be a much less assailable one than the other.

Now what will our readers think is the doctrine for the defence of which Dr. Paine so cunningly seizes upon the lungs as his citadel? Neither more nor less than this—that animal heat is a secretion; and, like other secretions, a production of pure vitality, to the parentage of which chemistry has no claim whatever. Here again we must revert his nomenclature. Secretion, as physiologists have always been accustomed to use the term, implies a *separation* from the circulating fluid of certain ingredients which preexisted in it; these ingredients being often previously united into the peculiar substance which is characteristic of the secretion (in which case their *mere* separation is most evident); or being so united in the very process of separation. However this may be, *the elements* must have preexisted in the fluid; and it implies the idea of an absolute creation of new matter in this act, to entertain any other opinion. Now, does Dr. Paine mean that *heat* was preexisting in the blood in a state not manifest to the senses, and that it is only *separated* by the act of secretion to which he attributes it? If so, we see no essential difference between his hypothesis and that of Dr. Crawford; since this preexisting heat exactly corresponds with the *latent caloric* of the chemist, which only requires certain chemical changes to render it sensible. On this view, then, Dr. Paine's hypothesis is only a modification of a form of the chemical explanation which has long been familiar. For any other, a new definition of the term secretion must be given; and we see no reason why the vital laws should not as readily *produce* gold or silver, as *secrete* heat from that which did not previously contain it.

In speaking of the relation between respiration and animal heat, we do not wish to be understood as asserting that this is direct or immediate. We believe it only to operate through the nutritive processes, and the molecular changes which they involve. The *aggregate* of these processes throughout the body may be most readily ascertained by comparing arterial and venous blood; and that the *principal* difference between these consists in the presence of oxygen in the former and of carbonic acid in the latter, cannot, we think, now be denied by any one who impartially views the evidence, and gives due weight to the causes that have produced those contradictory results which induce Dr. Paine to look with an eye of scepticism upon the whole. It is not, then, the change of arterial into venous blood, but the processes which cause that change, which are the real source of the production of heat. In these processes there are probably many chemical changes involved, besides the combination of carbon and oxygen; and it is to the aggregate of all these (the last, however, being the chief) that we attribute the liberation of caloric which was previously latent. These processes may, in some animals, continue for some time without a fresh admission of oxygen into the lungs; and in them, the dependence of animal heat upon respiration is therefore more remote than in others. But in insects, the feeble motion of whose blood is counterbalanced by the admission of air into every part of the system, the relation is extremely close. A stimulus which excites the individual to activity also increases the number of its respirations and its consumption of oxygen; and the temperature is raised, as Mr. Newport has shown, exactly in the same proportion. Now, in alluding to Mr. Newport's experiments, Dr. Paine entirely overlooks this fact; and hazards the bold assumption, that "upon this simple fact alone (the rapid increase of temperature when the insect is excited), so precise are the laws of nature—it will be safe to found a general induction as to the dependence of animal heat upon the forces of life," (p. 66,) that is, we understand Dr. Paine from his other remarks to imply its entire independence of all chemical laws. How this *general induction* from a *single fact* is to be attained, it surpasses our comprehension to discover; it savours a little of the "go-a-head" principle, which physiologists, of all men, should carefully eschew.

On one point, the connexion of the nervous system with the production of animal heat, we have pleasure in stating that we think Dr. Paine's views in the main correct and well expressed. We shall therefore present them in his own words.

"We have spoken of the probable controlling influence of the brain over the generation of animal heat, under particular circumstances. It may be more difficult to arrive at the extent of its influence in the natural state of the animal system. *Like other secreted products,* animal heat is, doubtless, primarily dependent on the organic powers. These are variously influenced by varying conditions of the cerebral and ganglionic systems; and, of course, their actions and the products of the secerning system, over which they preside, are affected in a corresponding manner, whilst they are also modified by the direct action of foreign agents. In the perfectly natural state, there is reason to suppose that the brain may have but little connexion with the phenomena, but may become powerfully instrumental in modifying the powers and actions and products of life, when unusual conditions exist, or when unusual impressions are transmitted to the brain. We shall see that analogy, as supplied by the vegetable kingdom, affords presumptive evidence that the brain may have no active participation in the elaboration of heat, in the natural condition of the body, whilst this induction is strengthened by what is known of other secreted products in both of the animated kingdoms. Still, in respect to the animal kingdom, the mere existence of the cerebral system, its remarkable properties and susceptibilities, and its intimate connexion with all parts of the organization, is, *primâ facie,* conclusive that it determines important influences upon the *vires vitæ* and that its presence is indispensable to the integrity of every function. This has been experimentally ascertained in reference to many; and that unusual or sudden impressions that are not unnatural, as the operation of the passions, for instance, may be extensively and profoundly propagated from the brain to other organs. It has been fully demonstrated (?) that the natural condition of the secretions depends upon the integrity of the nervous connexion betwixt the secerning organs and the brain; whilst it has been equally shown that the organic functions and all vascular action may be immediately and powerfully influenced by impressions made upon the brain." (Vol. ii. p. 23.)

In Dr. Paine's opinion, then, as in ours, the acknowledged influence of the nervous system upon the production of animal heat is exercised through the medium of the organic functions; and the difference between us consists in this, that *he* regards it as one of those functions incapable of being explained by any other than vital laws—which, in the present state of our knowledge of those laws, is equivalent to saying that we know nothing at all about it; whilst *we* consider it as a *result* of those functions produced by the molecular changes which they involve—these changes being themselves governed by the ordinary laws of chemistry, as we have elsewhere endeavoured to show.

In the last section of this essay, we find some original experiments on the heat of the stems of trees, made for the purpose of confirming John Hunter's results, and thereby proving that the evolution of heat is an organic function, and not dependent on the nervous system. Of Dr. Paine's qualifications as an experimenter, or rather, we should say, as a recorder of experiments, our readers may judge, when we tell them that, in his first series made at the beginning of April, he gives us a table of the degrees at which the mercury stood in thermometers inserted in the stems of twenty trees, varying from 48° to 68°, whilst, to enable us to compare these with the external temperature, he only tells us that the range of the thermometer in the shade, during the six hours that the experiments lasted, was from 38° to 52°, and that the temperature of a dead tree, selected as the standard of comparison, was 45° at the close of the experiments. Of a similar series, made ten days later, when vegetation was consequently more active, we are, in like manner, only informed that the range of the external thermometer, during five hours, was from 40° to 65°; the temperature of two dead trees at the end of the observation, 60°; and the temperature of the earth six inches below the surface 47° at the same period. Let our readers compare these, if they think it worth while, with the precisely-narrated experiments of Hunter, in which a distinct standard of comparison is given for every tree experimented on. In these it was often observed that the temperature of the stem was *lower* than that of the atmosphere, a result which Dr. Paine does not seem to have met with. This shows that there must be some cause interfering with the manifestation of the *proper heat* of the parts surrounding the bulb; and this cause we believe to be partly the slow conducting power of the wood in a transverse direction; and partly the important circumstance altogether overlooked by Dr. Paine, that the sap which ascends the stem is derived from a considerable depth of soil, (about four feet is stated as the average position of the spongioles of a large tree), and that its temperature is, therefore, no indication of the proper heat of the stem. Moreover, the vital process taking place in the interior of the stem are so very slight (the formation of new wood taking place just beneath the bark, and the old wood serving but as the conduit for the ascending sap) that this circumstance of itself indicates the fallacy of such experiments. We recommend to the attention of Dr. Paine, and of such of our readers as feel interested in the subject, the experiments of Dutrochet, made with the thermo-multiplier, in which the sources of fallacy we have indicated were carefully guarded against, and which are by far the most satisfactory to our minds of any of those which profess to prove that an evolution of temperature takes place during the *ordinary process of nutrition* in plants.*

The next essay professes to give the "Philosophy of Digestion;" by which we should understand a scientific account of the process, explaining it by the operation of known general laws of some description or other. This, however, is far from being the case. The author's object is to explode altogether the chemical theory; and to show that, once conveyed into the stomach, food is only acted on by the vital forces. Finding that our present knowledge of chemistry is insufficient to account fully for the reduction of the food, and its conversion into the organizable product albumen, he jumps at once to the conclusion that the gastric juice acts as a solvent in virtue only of the "vital forces" which are mixed up with it; and, as the separation of the fluid from the body does not prevent its retaining these, for a certain time, the solution which may be artificially produced by the employment of it is to be explained in the same manner. The experiments of Müller, Schwann, Eberle, and others, are very summarily disposed of; Dr. Paine proceeding upon the principle that if our present very imperfect knowledge of the principles of organic chemistry (a science confessed by all to be in its infancy) will not explain everything, those principles have *no share* in the result. The following is the commencement of his creed. "The vitalist maintains that the gastric juice is a substance *sui generis*, endowed with vital powers; that it can only be generated by a living stomach, and that it is liable to modifications which may arise in the vital condition of that organ; that it *cannot*, therefore, *be remotely imitated by art*, any more than art can manufacture semen or the cerebral substance; that its properties, like those of every other vital part, are in *perfect opposition* to the forces and agencies of chemistry," &c. &c. (p. 122). These "transcendental vitalists," as one of our contemporaries denominates them, seem quite to forget that there is no proof whatever that the gastric juice has any concern in *vitalizing* the alimentary substances submitted to its action. Its office seems to be to reduce them to the form of albumen, which is the chief constituent of the chyle as first absorbed. Now it is to the *fibrine*, which is *subsequently formed*, that the coagulation and other vital properties which the chyle possesses are due; and this substance, which Dr. Paine speaks of as existing in the stomach, is, we are tolerably confident, never found there, except in the state of half digested food. In fact, the gastric juice has for its office to reduce the aliment, which is to be presented to the absorbing origins of the lacteals, to the same state in relation to the animal, as the watery fluid, holding various substances in solution, conveyed by the soil to the roots of a plant, has to the vegetable which it sustains. There is no more reason to regard the one as a *vitalized* product than the other. The only indication of vitality in albumen is the globular form of its particles; and it is yet to be shown that this is not as characteristic of albumen as any crystalline form is of a particular salt. The organization and *consequent* vitalization of this substance commences as soon as, being taken into the vessels, it is admitted into the living system, to which, so long as it remains in the stomach, it is really external.

As connected with this subject, though by what train of thought is

* See B. and F. M. R. vol. ix. p. 546, and Ann. des Sci. Nat. N. S. Botan. tom. xii.

not very evident to us, we have an essay upon spontaneous generation, in which the author strongly opposes this hypothesis in all its forms. We are as convinced as he is, that no combination of inorganic elements can ever *spontaneously* produce a living organism, the previous agency of another living organized structure being a necessary condition of *their* organization. But we may take this opportunity of stating that our belief in the general proposition, which we long ago put forth in an interrogative form (vol. IV. p. 20), that "plants or animals of a high degree of organization are capable of producing from various parts of their tissues beings corresponding to those of the inferior orders of their kingdoms," has recently been much strengthened by additional evidence.

We have next a learned review of the Theories of Inflammation; the object of which is evidently to prove that they are all wrong; but it is not so evident what the author upholds as right, save that this disorder consists in disorder of the "vital forces." As far as we can understand his views, he regards inflammation as consisting in the exaltation of these forces. "The capillary vessels, then, performing at all times in health the actions of contraction and dilatation through vital forces, there can be no reason assigned why, when those forces are exalted, the contractions and dilatations should not be increased in a corresponding manner." (p. 156.) Having so recently discussed this subject in full, we shall not now enter upon it again; but we may remark that, by his almost exclusive attention to the state of the vessels, Dr. Paine seems to us to wander from the main point of enquiry, which concerns the relation of the blood and the tissues supplied. But, according to our author, although blood may be altered from its healthy condition, it never can be, strictly speaking, diseased; that is, it must always bear a *constant* relation to the solid tissues. This he endeavours to establish in his essay on Humoral Pathology, by what appears to us a most loose and inconsistent train of argument; to which it seems to us quite sufficient to reply that, in all cases of local disease, the blood must bear a *different* relation to the healthy and diseased solids respectively.

The next essay, professing to give the "Philosophy of Venous Congestion" is, we think, altogether the most valuable in the work; though we should be able to give it higher commendation, if it were compressed from *four hundred* pages to forty. It is marked by all the faults of the author's style of reasoning and writing; excessive dogmatism and exclusiveness, hasty inferences, vague statements, looseness of expression, and unnecessary amplification. But, withal, it contains some interesting views, which we deem worth prosecuting, although scarcely so well established by the author's arguments as he seems to consider them. By the term "venous congestion," Dr. Paine implies "a morbid fullness of the veins, arising from disease of their parietes; having no reference to the state of the arteries, and being wholly different from that fullness of the veins which is consequent upon a preternatural quantity of blood thrown upon them by the arteries under various influences. Nor do we recognize, in the last, as appertaining to this affection, that turgescence of the veins which is produced by obstructing causes." To establish the existence of such a condition as a primary morbid alteration, is the object of the present essay; and we think that the author is quite right in asserting that, in many instances, the enlargement of the veins and the stagnation of the blood in them cannot be accounted for on the ordinary theories, and that they are due to some change in the veins themselves. We are further disposed to admit the probability that this change is of an inflammatory character, though we cannot perceive any distinct proof of this. But we must protest against such a system of dogmatising as that which induces Dr. Paine to declare that, because *all* cases of venous turgescence cannot be explained by obstruction of the trunks or stagnation in the lungs, these causes never operate; for this in fact is his strange position. He refers to Goodwin, Bichat, Andral, Louis, Kellie, Broussais, and others, as having "clearly shown, either by experiment or by anatomical facts, that no pulmonary or thoracic affection can constitute an obstruction to the return of blood to the head." (p. 249.) Now let it be remarked that in this sentence he states the *impossibility* of the occurrence; and yet in the next, which he quotes from Broussais in support of his assertion, the *rarity* of it is alone intimated. "He remarked that the difficulty of the disgorgement of the superior vena cava of itself seldom produces cerebral symptoms." In the next page we are told that "if in dyspnœa the face be sometimes injected, or the jugulars swell, it arises frum compression of the superficial veins by the respiratory muscles, or, in the former case, a determination of blood upon the arterial system." Will this explanation account for that reflux of blood with every contraction of the heart, which causes the *venous pulse* so often observed in cases of obstruction in the pulmonary circulation, and which is permitted by the "safety-valve function" of the valves on the right side of the heart, when its cavities are over-distended? One would think, too, that Dr. Paine had never seen a case of asphyxia, in which the congestion of the cerebral veins, as well as of those of the face, liver, intestines, and general surface, is sufficiently apparent, and evidently depends upon the stagnation of the blood in the pulmonary capillaries. Dr. Paine will not, we apprehend, feel highly flattered by our comparing his mode of philosophising to that of Magendie; and yet, opposite as are their results, they are attained by processes essentially the same; each dogmatically condemning the theories of others, professing to abjure all theory, and yet building up hypotheses of his own still more unstable than those which he endeavours to overthrow. We will take just one more example: "In obliterations of the jugular veins, or of the vena cava descendens, there may take place preternatural accumulations of blood in the cerebral veins. But we do not find that they have ever been attended by any of the phenomena of congestion, or of extravasations of blood or serum, or by any interruption of the cerebral functions." (p. 252.) Surely *hanging*, whether judicial or suicidal, is not so rare an occurrence in America, that Dr. Paine has never witnessed its post-mortem phenomena; but if he is sceptical as to the disturbance of the cerebral functions which is stated on very credible authority to result from the obstruction to the venous circulation in the neck, he has only to place a ligature on any one in such a position that it shall compress the jugulars without interrupting respiration, and we are satisfied that he will be very soon desirous of loosening it.

Having established, in his own opinion, that "venous congestion is in no respect mechanically determined by a *remora* of blood," he proceeds to show, and we think with more success, that a mere passive relaxation of their parietes will not produce it; and that the cause is to be sought in some morbid condition of the trunks themselves. This he regards as of an inflammatory nature; producing varicose enlargements when local and chronic, and giving rise to "congestive fevers," of which the cholera asphyxia is one form, when general and acute. A considerable body of evidence, from symptoms, post-mortem appearances, and the effects of treatment, is brought forward in support of this view; and we are disposed to recommend it strongly to the attention of our readers, although not in all the exclusiveness in which it is urged by its author. We may remark that that kind of permanent congestion, or varicose condition of the veins of the choroid coat of the eye, which frequently produces well-marked changes in its external aspect, is unquestionably of inflammatory origin. Dr. Paine speaks highly of the effect of free venesection in the *early* stage of such forms of typhus as would otherwise *soon* require large quantities of stimulants to prevent fatal depression. In an appendix to this essay, the author attributes death by cold to *his* form of venous congestion, of which he considers that agent to be one of the most common exciting causes. We cannot but think, however, that he does not give sufficient weight to the congestion of the internal veins, which is produced by the contraction of those nearer the surface, and which, although *remotely* dependent upon the vital contractility of the superficial veins, is *immediately* brought about by a physical cause. May it not be that this mechanical distension is itself an exciting cause of the morbid condition of the parietes of the veins, to which Dr. Paine restricts the term congestion?

The next essay, "On the comparative Merits of the Hippocratic and Anatomical Schools of Medicine," contains many sound remarks on the absurdity of the pretensions of the modern French school of morbid anatomy, and the necessity of the observation of the phenomena of disease in the living state for the success of medical practice. Having on several occasions expressed our opinion strongly on this point, both in its practical and abstract bearings,* we shall not now go over the ground again with Dr. Paine; but we may remark that he does not, in our opinion, sufficiently discriminate between the *science* and *art* of medicine — pathology and practice. Hence, if a certain fact, or series of facts, is useless as regards the latter, he neglects its application to the former; forgetting that the more perfect the science is made the more sure must the art ultimately become. He seems to forget, also, how much the observation of phenomena during life has been guided by the knowledge derived from post-mortem examination, and how frequently the value of a particular sign would be lost, if it were not connected in the mind of the practitioner with a coincident morbid state, ascertained by post-mortem examination of similar cases to be probably existing. How else, for example, should we have learned that the occurrence of delirium in acute rheumatism is an almost certain indication of incipient disease of the heart, and that remedies must be promptly applied to the chest instead of to the head? Our readers will, doubtless, think of many similar instances for themselves. It is an old observation, that we do not know the value of a blessing until we have lost it; and we would say, in like manner, that we could scarcely tell how much of our knowledge

* For the latter, see especially vol. VI., p. 108.

of the phenomena of disease in the living state is really due to post-mortem anatomical researches, unless we were entirely deprived of the assistance we have derived from that source.

The work concludes with a severe review of the writings of Louis, in which Dr. P. points out certain alleged fallacies of his method of generalizing, and exposes his hasty condemnation of previous observers. It may be thought a little strange that Dr. Paine should see these faults so glaringly in another, but should be so utterly unconscious of them in himself; but, alas! for human nature, such a self-delusion is by no means uncommon. We consider this review as strongly marked as any of Dr. Paine's writings by his peculiar exclusiveness; for, not being able to allow to M. Louis's system of observation all the merit which has been claimed for it, he seems to regard it as not only useless but strongly injurious; and he does not give him by any means sufficient credit for the mass of valuable materials which he has collected for the benefit of those who can use them aright, or for the numerous and admirable results, pathological and practical, which have flowed from the use of his method by himself and followers.

In taking our leave of Dr. Paine, we have to express our regret that it has been our misfortune to differ so materially from him in our estimate of the value of his opinions. We doubt not that none but the most laudable motives could have induced him to bestow so much labour and expense on the propagation of these opinions, and we feel grateful to him for the frequent and respectful appeals which he makes to our sentiments; but we cannot blind ourselves to the want of adaptation between the greater part of his volumes and the present state of medical science, and to the frequency of the rash assumptions which prevent us from attaching weight to his judgment. Learning, zeal, and industry they certainly evince, and those of our readers who find these qualities sufficiently attractive, and who can spare time to discuss fifteen hundred full octavo pages, had better form their own opinion of the other merits of the work, without being influenced by our award.

---

